# Case of Wound of the Liver

**Published:** 1873-09

**Authors:** G. W. Stewart

**Affiliations:** Muscatine, Iowa


ARTICLE II.—*Case of Wound of the Liver.* By G. W. STEWART, M.D., Muscatine, Iowa.

Anthony B., a lad aged 17, while standing beside a plow team, with the reins about his neck, had his team attempt to run off, and before he could stop them he was thrown on his face in front of the plow, the point of which, engaging him in the region of the small of the back, and dragging him for some distance, inflicted the following described wound:

A semicircular incision in the left hypochondriac region, beginning at a point over the fourth lumbar vertebra, passing first forward and upward, thence backward to a point opposite to and about one and a half inches from the first lumbar vertebra, measuring at the surface, eleven inches; penetrating the integument, passing through aponeurosis of transversalis muscle, latissimus-dorsi, sacro-lumbalis, longissimus-dorsi, serratus-posticus-inferior, and quadratus-lumborum muscles, engaging the twelfth rib and denuding it of its periosteum in its anterior or outer third; passing through the peritoneum and entering the liver at the left lateral ligament, passing inward, forward and upward, in a direction toward but stopping just short of the intersection of the longitudinal with the transverse fossa, within which, and only a few lines beyond the terminus of the incision, could be distinctly felt the hepatic artery. Nearly or quite all of that portion of the left lobe of the liver anterior to the incision in its substance as described, we found protruding through the wound.

We saw the case about two hours subsequent to the occurrence of the accident, and in addition to the particulars already given, found our patient and his wound in the following condition:—Patient nearly comatose from loss of blood; hemorrhage, however, being from small vessels only, had ceased. The integument bordering the wound, was very much retracted, as were also the muscles through which it (the plow) passed, except the latissimus-dorsi at its inferior border, where, from the direction of the incision, the muscular fibres were simply separated—thus presenting a wide and gaping wound.

Upon passing our hand into the abdominal cavity, we found present a large amount of blood, which we removed with a sponge, and continued our search for more extended injuries than those already described; examined the stomach, which, being empty, and in a partial state of collapse, had escaped injury; as had also the intestines, diaphragm and other organs. The abdominal aorta could be distinctly felt in the floor of the wound, but uninjured.

TREATMENT.—Seizing that portion of the liver protruding from the wound, we replaced it as nearly as possible in its normal position; but upon removing our hand it came immediately out again, seeming to require considerable pressure to maintain it in place; we returned it a second time, and employed an assistant to retain it in position until we could close up the wound, and apply over it a compress to hold it securely in position.

The periosteum of the twelfth rib being only split, we gave it but little attention, thinking that it would take care of itself; and hemorrhage having, as we said, ceased, we proceeded to close up the wound; in this we experienced some difficulty in consequence of the extensive laceration of tissues, but finally succeeded, and finished the dressing by placing over that portion of the liver which had been displaced, a large compress, and over that and around the body several times, a wide roller bandage.

We then directed that cold water be applied to the wound every fifteen minutes, which we continued for ten days, making no changes during that period, other than removing, after the lapse of twenty-four hours, the compress, the pressure of which we feared might induce inflammation, and on the third day beginning the use of carbolic acid, a weak solution of which we applied once every hour.

Fearing peritonitis, and expecting it to manifest itself within two or three days, we were very sparing of diet, allowing our patient for some days only articles of the blandest character. Administered on the second day, that he might obtain sleep, an opiate, with the direction that in case of the occurrence of pain in the wound or its vicinity, there be given every hour or two a powder containing a grain of pulverized opium.

On the sixth day the wound began to discharge pus quite freely, and mixed with it was a greenish substance that we took to be bile, the latter of which was kept up for a period of two weeks.

Our patient gained strength from the first, pulse becoming from day to day more firm; the wound, which we dressed daily, looked healthy, and, what is remarkable, there *never appeared at any period the first indication of peritonitis.*

On the twelfth day danger of inflammation being passed, or less imminent, we began to apply less frequently our cold water, applying it and the carbolic acid every hour, and also to feed our patient more liberally.

At this time the flaps sloughed off, and exposed to view the liver, and also the twelfth rib, the former of which we discovered to be protruding somewhat through the wound, a portion equal in measurement to a two inch cube being external to the quadratus-lumborum muscle; we endeavored to reduce it as at first, but discovered obstacles in the way; adhesions had formed between the walls of the abdomen and both the superior and inferior borders of the left lobe, which rendered that lobe immovable.

It being impossible for the wound to heal in this condition, we resorted to the use of caustics and the knife, (using the caustic potash), for the purpose of getting rid of this protruding portion of the liver, applying the caustic daily, and afterward paring off with the knife cauterized material, until the prominence had disappeared; having in readiness the actual cautery to use in case of troublesome hemorrhage, should we go too deep with the knife, but no hemorrhage sufficient to occasion alarm occurred.

We next examined our rib denuded of periosteum, and discovered that it was not taking care of itself so nicely as we anticipated it would, and after watching it a few days, decided that amputation would be necessary, as it was becoming still more denuded, and besides was standing out more prominently than it should, and irritating the wound, preventing it from healing kindly; therefore with bone forceps we removed about one-half of it. This gotten rid of, the wound improved in appearance; the two fragments of liver united very perfectly as we could discover, being able to see the line of union in a part of its course; the cauterized surface healed speedily, and granulations sprang up rapidly in the muscular tissue, continuing until the divided muscles were reunited and integument formed over the entire surface.

The wound was inflicted May 7, 1873, and can be said to have terminated in complete recovery in about seventy days time—a result to us unexpected. We have searched for and examined histories of cases reported of a similar nature, and although there may have been recoveries in cases of a like character and as severe, we have not yet found them. Recoveries are reported, but none whose histories we have seen in which the injuries were so extensive.

